# The mediating effect of job satisfaction and emotional exhaustion on the relationship between psychological empowerment and turnover intention among Chinese nurses during the COVID-19 pandemic: a cross-sectional study

**DOI:** 10.1186/s12912-023-01357-y

**Published:** 2023-06-27

**Authors:** Jinliang Ding, Yufang Wu

**Affiliations:** 1 School of Humanities and Teacher Education, Wuyi University, Wuyishan, 354300 China; 2 School of Psychology, Fujian Normal University, Fuzhou, 350000 China; 3 School of Economics and Management, Ningde Normal University, Ningde, 352100 China

**Keywords:** Psychological empowerment, Job satisfaction, Emotional exhaustion, Turnover intention, Nurses, COVID-19 pandemic

## Abstract

**Background:**

Turnover intention occurs frequently in nurses and psychological empowerment has been shown to be major factors that influence turnover intention. However, little is known about the driving force behind turnover intention among nurses in China during the COVID-19 pandemic.

**Objectives:**

To investigate the mediating role of job satisfaction and emotional exhaustion on the association between psychological empowerment and turnover intention among Chinese nurses during the COVID-19 pandemic.

**Methods:**

A cross-sectional design was conducted in China. A total of 507 nurses completed scales of psychological empowerment, job satisfaction, emotional exhaustion and turnover intention anonymously. Descriptive analysis, Pearson’s correlation analysis in SPSS 23.0 and structural equation modeling (SEM) by Mplus 7.4

**Results:**

Psychological empowerment had a significantly effect on turnover intention through three significantly indirect pathways: (1) through job satisfaction (B = -0.14, SE = .03, 95% CI = [-.19, -.09]). (2) through emotional exhaustion (B = -0.07, SE = .02, 95% CI = [-.11, -.03]). (3) through the chain mediating effect of “job satisfaction → emotional exhaustion” (B = -0.12, SE = .02, 95% CI = [-.16, -.09]).

**Conclusions:**

Intervention measures to reduce the incidence of turnover intention of nurses should include the evaluations of work demands and emotional exhaustion of nurses and organization’s management strategies to promote their psychological empowerment and job satisfaction.

**Supplementary Information:**

The online version contains supplementary material available at 10.1186/s12912-023-01357-y.

## Introduction

*Turnover intention* refers to the process whereby employees deliberate over withdrawal from the current job and consider for other jobs within a certain period [1]. Some studies estimate that 30.5% doctors in South Korea reported intend to leave their jobs [2], and about 30% to 40% of teachers leave the profession within their first five years [3]. One survey showed that the proportion of nurses with clear turnover intention ranged from 20.2 to 56.1% [4], and this is highest compared to some other professions, showing that the body of nurses is unstable and constantly in a state of change [5].Turnover intention is the antecedent variable of turnover, which can predict actual turnover rates [6]. Recent studies suggested that the high rate of nurse turnover not only affected the care quality, but it also brought severe economic losses to these countries [7], including productivity loss and costs of hiring and training new staff [8], especially in remote and poverty-stricken rural areas of China, where nurses are in short supply [9]. Therefore, the study of turnover intention is helpful for organizations concerned with taking preventive measures to reduce eliminate the nurse intention to leave before actual turnover occurs.

Based on the previous literature on this topic, psychological empowerment, job satisfaction and emotional exhaustion have become the focus of academic attention. Spreitzer [10] defined psychological empowerment as a type of motivational state that is achieved through the combined effects of four cognitions: meaning, competence, self-determination, and impact, reflecting that employees are interested in and feel capable of shaping their work environment [11]. Decades of research have shown that psychological empowerment facilitates one’s working condition (e.g., professional identity, job involvement, and job satisfaction) to escape work burnout and alleviate turnover intention [12]. Job satisfaction refers to the overall view and feelings of employees about their work [13], which can contribute to reducing work burnout and turnover intention of employees [14, 15]. Moreover, emotional exhaustion, as the core dimension of burnout, is related to negative psychological syndrome, which in turn would lead to turnover intention [3].

Yet, surprisingly, most of these studies were conducted in a piecemeal fashion, for example, two studies examined the relationships between job satisfaction, burnout and turnover intention in a group of medical workers [9, 14]. Boudrias et al. showed the role of psychological empowerment in the reduction of burnout in healthcare workers [16]. There is a paucity of research examining the specific relationships among psychological empowerment, job satisfaction, emotional exhaustion and turnover intention, especially using a sample of nurses in China, who are often regarded as a high-risk group of work burnout and turnover intention. Moreover, in a Western context, concepts such as psychological empowerment have been successful not only in inspiring employee loyalty but also in improving performance [17, 18]. Psychological empowerment takes different forms for different people and can vary by context. It is a dynamic variable that has been proven to fluctuate across cultural boundaries [19]. Thus, it is necessary to expand the meager body of research providing empirical studies of psychological empowerment and work attitude-related variables affecting nurses in China. Therefore, we have tried to examine the link between psychological empowerment and turnover intention, and identify the mediating roles of job satisfaction and emotional exhaustion under this relationship among nurses.

### Psychological empowerment and turnover intention

According to the Cognitive Empowerment Model, psychological empowerment is the cognitive evaluation of work [20], such that individuals with high psychological empowerment tend not only to positively evaluate their work environment, but also demonstrate better work performance, the result of which is greater loyalty and emotional attachment to their organization. Such individuals are less likely to consider quitting their jobs than are people with lower levels of psychological empowerment [21]. As explained by Spreitzer [10], psychological empowerment relates to how competent people feel in an empowered work environment. Those who feel more confident about their ability to perform their work successfully tend to be more interested in that work and experience greater job satisfaction. This effectively increases their sense of belonging to an organization, making them less willing to leave [22]. Prati and Zani noted that when healthcare professionals feel empowered, they are more likely to stay at their organization rather than leave it [12]. In contrast, a lack of perceived empowerment means employees feel as if they have lost control over management of their resources, creating a definite sense of job insecurity and resulting in a desire to escape their current occupation [18, 23]. Therefore, we propose the following hypothesis:

*Hypothesis 1*: Psychological empowerment is negatively related to turnover intention.

### The mediating role of job satisfaction

*Job satisfaction* has been described as the degree to which employees have a positive affective orientation towards employment by an organization [24]. According to the Cognitive Evaluation model of psychological empowerment, psychological empowerment is the cognitive process of evaluating one’s work [20]. When an individual's perception of their level of empowerment is high, their work can bring them meaning and influence, possibly increasing their internal motivation and sense of self-efficacy and autonomy, and in turn promoting satisfaction with their work [20]. Studies have also documented that psychological empowerment is associated with job satisfaction positively [25–27].

On the other hand, many turnover models regard job satisfaction as an important predictor of turnover intention, arguing that individual dissatisfaction with their current positions can cause a number of serious adverse reactions and eventually lead to an intention to leave [28, 29]. As the Turnover Decision-Making Process Model proposed by Mobley et al. suggested, employees regularly evaluate their current job and engage in careful consideration before resigning their position. If they are very dissatisfied, they may consider leaving and then evaluate the cost of finding a new job [28]. Conversely, individuals with a high level of job satisfaction typically have a positive attitude towards their work and are more likely to adopt a positive approach to completing work-related tasks [30]. Studies have showed that nurses with low job satisfaction were more likely choose to leave their positions [31, 32]. On the foundation of these findings, the following hypothesis is proposed:

*Hypothesis 2*: Job satisfaction mediates the relationship between psychological empowerment and turnover intention.

### The mediating role of emotional exhaustion

Emotional exhaustion refers to the draining of personal resources or overuse of one’s psychological and emotional resources, which is a core dimension of burnout [33]. A large number of studies have proved that psychological empowerment may predict emotional exhaustion [34, 35], this can also be supported by the Job Demands- Resources Model proposed by Demerouti et al. in 2001, which states that Burnout is likely to occur when employees are unable to handle properly the imbalance between job resources and work demand. Individuals with high psychological empowerment have psychological resources sufficient to meet their needs and alleviate unhealthy emotions caused by a shortage of work resources [36]. In contrast, individuals with low psychological empowerment fail to effectively detach from work [37], and an overload of work demands can result in a state of fatigue, often leading to emotional exhaustion [38]. It is thus clear that high level of psychological empowerment corresponds to low level of emotional exhaustion.

In addition, previous studies have shown that emotional exhaustion also relate with turnover intention positively significantly [14, 39, 40]. First, the Conservation of Resources Model indicates that exhausted employees consume work resources excessively. Once personal resources cannot be supplemented in a timely fashion, they suffer a corresponding loss spiral that can eventually lead to higher turnover [41]. Second, according to the Social Exchange Theory, all human behavior is dominated by the exchange of awareness activities. Individuals engage in avoidance or withdrawal-related coping strategies (such as forming an intent to quit) when they feel unfairly penalized by their organization, in an effort to lessen the psychological cost of emotional exhaustion [42], and protect themselves from further damage [43]. Therefore, integrating the antecedent and effect variables of emotional exhaustion, the following hypothesis is proposed:

*Hypothesis 3*: Emotional exhaustion mediates the relationship between psychological empowerment and turnover intention.

### Mediating roles of job satisfaction and emotional exhaustion

Both job satisfaction and emotional exhaustion are associated with turnover intention. In addition, studies have found that job satisfaction and emotional exhaustion are related [15, 44]. This suggests that job satisfaction and emotional exhaustion may influence one another and then contribute to decreasing turnover intention. If so, two potential mediate effects should be considered. One possibility is that individuals with low job satisfaction will increase the level of emotional exhaustion, which in turn contributes to an increase in individuals’ turnover intention. The other is that emotional exhaustion increases turnover intention through job satisfaction. The former seems plausible because evidence proves that low job satisfaction might lead to work stress and occupational burnout [15]. What’s more, in a study examining the role of emotional exhaustion in the relationship between job satisfaction and turnover intention, Scanlan and Still found that individuals with high job satisfaction who have the positive affective orientation towards organization will help avoid the depletion of emotional exhaustion, thus affecting a rung of behaviors such as turnover intention [14]. These findings suggest that it is more reasonable to treat job satisfaction as a factor affecting emotional exhaustion than to do the opposite. Therefore, it is likely that psychological empowerment may be a predictor of job satisfaction, and job satisfaction may influence emotional exhaustion and decrease turnover intention. Thus, we propose:

*Hypothesis 4*: Job satisfaction and emotional exhaustion play a chain mediating effect in the association between psychological empowerment and turnover intention.

### Aims

**Fig. 1:**
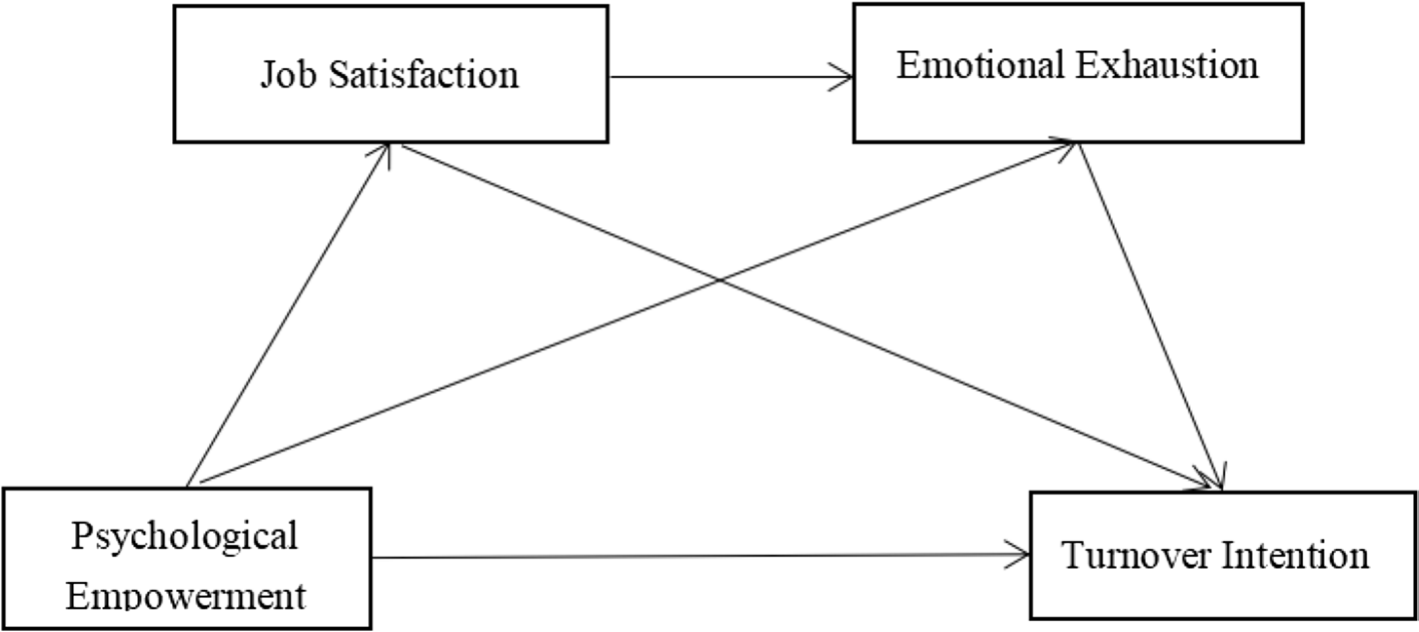
The proposed multiple mediation model

Turnover intention in nurses was reported more during outbreaks COVID-19, which has become an obstacle in the development of the healthcare system [45]. The high rate of turnover intention among nurses during the spread of infectious diseases can lead to unsatisfactory patient expectations and lower patient satisfaction, thus significantly affecting the provision and quality of health services and facility management [46]. There is evidence that the low retention of nurses in healthcare practices is related to heavy workload, burnout and job dissatisfaction [47]. Therefore, the purpose of this study is to propose and test a serial multiple mediator model (see Fig. 1) to analyze the influencing factors of nurses’s turnover intention during COVID-19 pandemic in China, to provide an objective basis for preventing the turnover phenomenon in the context of COVID-19.

## Method

### Design and participants

A convenience sampling method was used to recruit nurses from different hospital in 8 cities, including Fuzhou, Guangzhou, Nanping, Longyan, etc., from March to July 2022. Inclusion criteria of the study were more than six months of clinical work experience and a willingness to participate in the study. The exclusion criterion was if more than half of the items were answered in a repetitive fashion, or nonconforming questionnaires (answer time less than 100 s). Equation *N* = 4Uα^2^S^2^/δ^2^ was used to calculate the sample size [48]. S = 0.51 is calculated from the presurvey, the allowable error δ is set to 0.1, and α is set to 0.05, so *N* = 4 × 1.96^2^ × 0.51^2^/0.1^2^≈400. Considering the possibility of invalid questionnaires, we distributed a total of 530 questionnaires. Finally, after 23 unqualified questionnaires were deleted, 503 results were included in the analysis. In addition, Bentler and Chou proposed that the sample size should be more than 10 times the observed variables [49]; hence, a sample size of 503 met the requirement for testing the hypothesized models.

### Procedure

Nurses from different hospital were invited to participate in this study on a voluntary basis. The whole procedure was completed through an online questionnaire system called Wenjuanxing. With the cooperation of hospital authorities, the questionnaires were sent out in electronic form and participation were anonymous and voluntary. We sent the participants an online link to the survey which contained the scales and consent beginning the survey. The questionnaire required approximately 10–15 min of completion time. All participants who completed the survey get a chance of online lottery after completing questionnaires. The study was approved by the Ethics Committee of the Wuyi University.

### Measurements

#### Demographic information

A demographic questionnaire assessed the participants’ characteristics including gender, area, education levels, working experience and professional title.

### Psychological empowerment scale

The 12-item Chinese version of Psychological Empowerment Scale revised by Li et al. comprises four dimensions: work meaning, autonomy, self-efficacy, and work impact [50]. Items (e.g.,“What I have done at work is meaningful to me.”) are rated from 1(strongly disagree) to 5(strongly agree). Higher scores indicate higher levels of psychological empowerment. The validity of the scale has been verified in Chinese research (χ^2^ /df = 1.89, RMSEA = 0.04, CFI = 0.98, NFI = 0.95, GFI = 0.98, IFI = 0.98, IFI = 0.98), and the Cronbach’s alpha of four dimension was 0.76, 0.73, 0.69, 0.75, respectively [50].

### Emotional exhaustion scale

The five-item Chinese version of Maslach Burnout Inventory-General Survey developed by Li et al. was a 7-point Likert scale, ranging from (1 never) to 7 (every day), indicating their feelings of emotional exhaustion [51]. An example items is “Working all day is really tired for me”. Higher scores indicate a higher level of emotional exhaustion. The scale has been found to have a strong convergent validity (χ^2^ /df = 3.99, RMSEA = 0.08, CFI = 0.91, NFI = 0.89, GFI = 0.91, IFI = 0.92, IFI = 0.92) and sound reliability (the Cronbach’s alpha was 0.88) [52].

### Job satisfaction scale

The job satisfaction questionnaire compiled by Schriesheim and Tsui consists of six items, requiring individuals to report their satisfaction with work intensity and pressure, relationship with leaders and colleagues, salary, promotion and development opportunities [53]. Items are rated on a 5-point Likert scale (1 = very dissatisfied, 5 = very satisfied), with the higher score suggesting more job satisfaction. The questionnaire is widely used to measure job satisfaction in China, with good reliability (the Cronbach’s alpha was 0.78) [54].

### Turnover intention scale

The 3-item Turnover Intention Scalewas a 7-point Likert scale, ranging from 1 (never) to 7 (very frequently), with higher scores indicate stronger turnover intention [55]. A sample item is “I've been thinking about resigning recently.” The scale has been widely used in the Chinese population, with acceptable reliability (the Cronbach’s alpha was 0.78) [56].

### Statistical analysis

First, Harman’s single-factor test was conducted to assess the possibility of common method bias, and the results show that the interpretation rate of the first factor was 10.17%, less than 40%, indicating that there was no common method variance in this study [57]. Then, descriptive statistics and Pearson’s correlation analysis were performed with SPSS 23.0. Last, Structural equation modeling (SEM) procedure using the maximum likelihood estimate (MLE) was employed to examine the serial multiple mediation model by Mplus 7.4. The following criteria were used to evaluate fit: the value of χ2 /df is smaller than 5, CFI and TLI values are above 0.90, and the RMSEA and SRMR values are equal to or smaller than 0.08 [58]. Finally, bias-corrected bootstrapping was used to examine the indirect effect, with a 95% bias-corrected confidence interval (CI) for the indirect effect based on 5,000 bootstrap samples [59]. Gender, area,working experience, professional title and educational background were controlled as covariate variables in the model. The level of significance was set at *p* < 0.05.

## Results

### Characteristics of participants

As shown in Table 1. Among the total sample 12.4% was male, with 24.3% being from the low-risk COVID-19 area.The average age was 31.44 (SD = 1.69), ranging from 22 to 45 years old.

**Table 1:** Demographic of respondents

	*n*	%
*Gender*		
Male	63	12.4
Female	444	87.6
*Age, M* ± *SD*	31.44 ± 1.69	
*Education level*		
technical secondary education	117	23.1
Junior college	198	39.1
Undergraduate and above	192	37.9
*area*		
low-risk COVID-19 area	123	24.3
medium-risk COVID-19 area	182	35.9
high-risk COVID-19 area	202	39.8
*Professional title*		
Junior	296	58.4
Intermediate	143	28.2
Senior	68	13.5
*Working experience*		
Less than 5 years	87	17.2
6–10 years	99	19.5
11–15 years	89	17.6
16–20 years	89	17.6
More than 20 years	143	28.2

### The prevalence of turnover intention

The score of Turnover Intention Scale is divided as binary variable in order to see the incidence more intuitively. If the total score greater than or equal to median value (6) indicates that turnover intention exists. The prevalence of turnover intention was 65.3%. The prevalence of turnover intention in different area is different (χ2 = 14.88, *p* < 0.001), specifically, the nurses in high-risk areas reported significantly higher turnover intention (73.8%) than those in medium-risk areas (64.36%) (χ2 = 4.04, *df* = 1, *p* < 0.05) and low-risk areas (52.8%) (χ2 = 14.87, *df* = 1, *p* < 0.001), and the nurses in medium-risk areas reported significantly higher turnover intention (64.36%) than those in low-risk areas (52.8%) (χ2 = 3.99, *df* = 1, *p* < 0.05).

### Descriptive, reliability and correlational analysis

As shown in Table 2. Psychological empowerment was positively related to job satisfaction, while negatively related to emotional exhaustion, and turnover intention, respectively. Job satisfaction was negatively correlated with emotional exhaustion, and turnover intention. Finally, emotional exhaustion was positively related to turnover intention. Four measures have good reliability in current study.

**Table 2:** Descriptive, reliability and correlational analysis

Variable	*M* ± *SD*	1	2	3	4	Cronbach’s alpha
1. psychological empowerment	3.94 ± 0.63	-				0.91
2. job satisfaction	3.54 ± 0.74	0.54^***^	-			0.88
3. emotional exhaustion	2.92 ± 1.13	-0.36^***^	-0.50^***^	-		0.94
4. turnover intention	2.37 ± 1.19	-0.35^***^	-0.54^***^	0.69^***^	-	0.86

*N* = 507. ^***^p < 0.001

### Mediational analysis

**Fig. 2:**
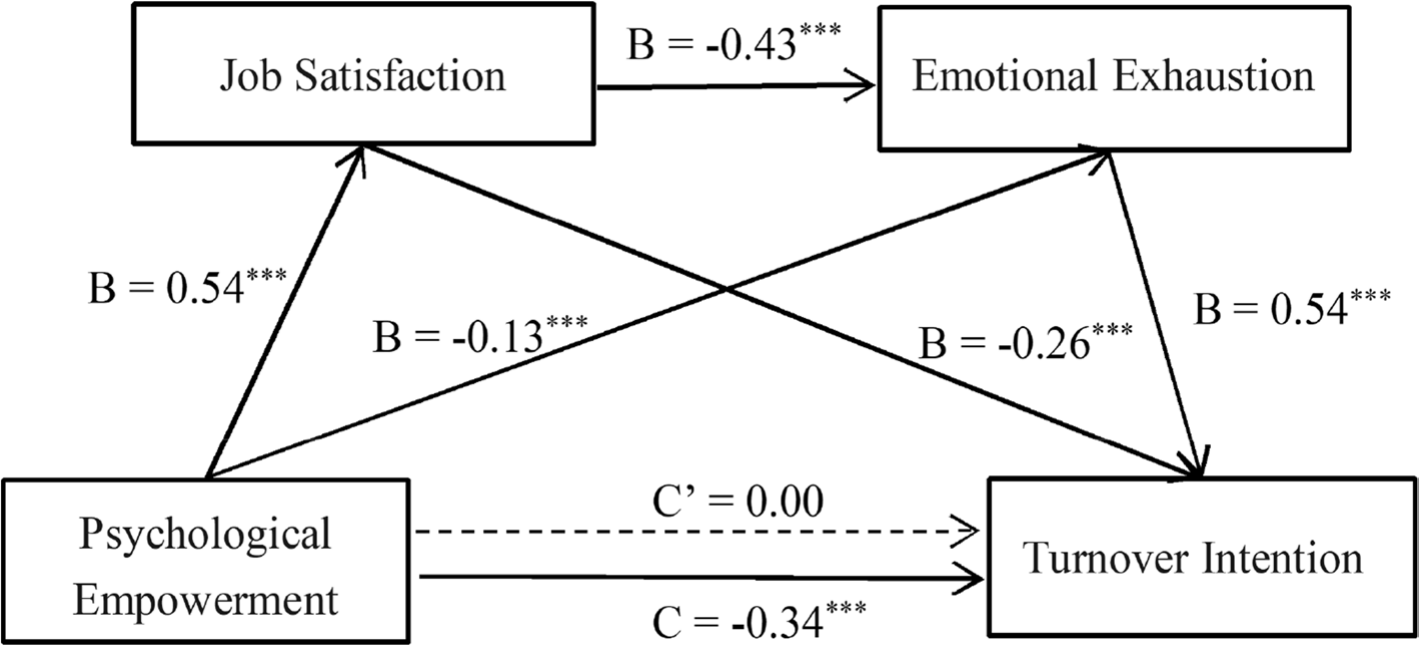
The hypothesized model. B represents the direct effect coefficient of the antecedent variable on the outcome variable. C’ represents the direct effect coefficient of psychological empowerment on turnover intention, and C represents the total effect coefficient of psychological empowerment on turnover intention. All path coefficients were standardized,.^***^p < 0.001

The results of structural equation model showed that the hypothesized model provided a good fit to the data (2 /df = 4.14, *p* < 0.001, RMSEA = 0.07, SRMR = 0.04, CFI = 0.95, TLI = 0.90). As shown in Fig. 2, the total effect of psychological empowerment on turnover intention was significant (B = -0.34, *p* < 0.001), but the direct effect was found to be non-significant after controlling the impacts of job satisfaction and emotional exhaustion (B = 0.00, *p* > 0.05). Therefore the effect.of psychological empowerment on turnover intention was totally mediated by job satisfaction and emotional exhaustion.

The Bootstrap results (see Table 3) showed that the total indirect effect of psychological empowerment on turnover intention was -0.34. Specifically, the indirect effect of psychological empowerment on turnover intention via job satisfaction and emotional exhaustion was -0.14, -0.07, and accounting for 41.18% and 20.59% of the total effect, respectively. Besides, the indirect effect of psychological empowerment on turnover intention via the job satisfaction and emotional exhaustion successively was -0.12,accounting for 35.29%. Taken together, except H1, H2, H3, and H4 were supported.

**Table 3:** Bias-correlated bootstrap estimate of the Standardized indirect effect for the proposed model

Paths	Estimate value (standardized)	*S.E*	Bootstrapping 95% CI		Account for the total effect (%)
			*Lower*	*Higher*	
Total effect	-0.34	0.05	-0.42	-0.25	
Direct effect	0.00	0.04	-0.07	0.07	
Total indirect effect	-0.34	0.04	-0.41	-0.27	
1. PE → JS → TI	-0.14	0.03	-0.19	-0.09	41.18
2.PE → EE → TI	-0.07	0.02	-0.11	-0.03	20.59
3.PE → JS → EE → TI	-0.12	0.02	-0.16	-0.09	35.29

*Abbreviations: PE* Psychological empowerment, *JS* Job satisfaction, *EE* Emotional exhaustion, *TI* Turnover intention

## Discussion

In this study, the prevalence of turnover intention of nurses during the COVID-19 epidemic were investigated. Based on previous studies, the relationships between psychological empowerment, job satisfaction, emotional exhaustion and turnover intention were examined, and serial mediation models were constructed.

We found that the prevalence of turnover intention were 65.3%, which higher than other countries in normal time (e.g. 35.5% of nurses in Italy) [60]. This is consistent with previous findings that turnover intentions of healthcare workers during COVID-19 were higher than turnover before COVID-19 [45]. The results showed that the nurses in high-risk areas reported significantly highest turnover intention. Nurses, the main force in the fight against the pandemic, are at greater risk than others [61], multiple studies showed that 76.9% and 52% of healthcare workers had low willingness to work due to lack of proper protective equipment, long working hours, high infection rates and pressure lead to nurses’ turnover intention during COVID-19 period [62, 63].

This study found that the psychological empowerment didn’t significantly predict the turnover intention, not supporting H1. This finding is not consistent with most previous studies [34, 35]. The results indicated that psychological empowerment can fluctuate across cultural boundaries and vary by context [19]. Individuals from high power distance cultures, like China, a country categorized as a collective and high-power distance culture [64], are used to working in centralized environments where decisions and task guidelines are provided from above and overall the work environment is formal [65]. As a result, it is possible that they may perform better in disempowered conditions where tasks are structured, and responsibilities are explicit [19]. Conversely, they may not perform as well when placed in an empowerment intervention in which they are required to work with more responsibilities, respond to more information, and work autonomously. Perhaps this is the reason for this phenomenon psychological empowerment didn’t predict directly turnover intention.Nonetheless, follow-up studies are necessary to incorporate cultural differences when assessing psychological empowerment, and to further investigate the impact of power distance orientation on empowerment and related areas such as turnover intention.

The current study revealed that the mediating role of job satisfaction in the association between psychological empowerment and turnover intention. First, this finding is in line with previous results that suggest a positive link between job satisfaction and turnover intention [14, 15]. According to the Turnover Decision-Making Process model [28], job satisfaction is the first discovered attitude variable that affects employees' turnover, employees' dissatisfaction with their work will lead to the idea of resignation. Previous studies have found that job satisfaction has a negative impact on turnover intention in China [30, 66]. Moreover, these results agree with the cognitive evaluation model, which posits that psychological empowerment is the intellectual assessment of work produced by individuals integrating their own and others' views into work environment-related events [20]. Researchers have found that individuals with higher (vs. lower) psychological empowerment tend to evaluate their work more positively and are unwilling to leave jobs they enjoy; thus, psychological empowerment helps reduce the turnover rate [12, 20]. By contrast, individuals with low psychological empowerment have lower job satisfaction and higher turnover intention [67].

The study also verifies emotional exhaustion mediates the effect of psychological empowerment on turnover intention. Similar to earlier research, the present study found that psychological empowerment and EE are key factors connected with turnover intention [39, 43, 44].The results are in line with the Conservation of Resources Model, exhausted employees deplete personal resources that cannot be regenerated by psychological empowerment. This tends to produce undesirable consequences such as the formation of an intent to quit. A higher psychological empowerment level means more job resources [10, 20], which in turn helps reduce psychological pressure, the symptoms of emotional exhaustion [68], and turnover intention [69]. By contrast, individuals with lower level of psychological empowerment have a lower sense of identity with work and consequently felt higher level of emotional exhaustion and intent to quit.

As predicted, the serial mediation analysis results showed that the relationship between psychological empowerment and turnover intention through a process in which high psychological empowerment individuals have an increased tendency to be more job satisfaction, resulting in less emotional exhaustion and translating into a decreased turnover intention. This result follows studies highlighting that a sense of job satisfaction is an important factor in an individual’s feeling of emotional exhaustion and level of turnover intention [14, 15]. In contrast to previous research, this study reveals that psychological empowerment can be associated with turnover intention through multiple pathways. The results of the current study guide on how to intervene in the turnover intention of low psychological empowerment nurses.

The results of this study have important theoretical significance and practical value for reducing the turnover intention of nurses during the COVID-19 pandemic. To reduce the risk of turnover, the following recommendations are made. First, for manager of nurses, officials should aim to improve nurses' psychological empowerment by strengthening their professional knowledge and discourse power, and curbing turnover intention at the source. Second, we also recommend that the officials in medical departments pay attention to enhancing nurses' job satisfaction by improving the structure of hospital culture and enhancing nurses' professional autonomy. Different administrative measures and means of stimulation ought to be.adopted aimed to nurses' needs. More importantly, attempts should be made to improve the organization' s management and increase job satisfaction, including by designing a performance-based salary structure, encouraging participation in decision-making, highlighting attractive job opportunities, and providing safe working conditions and information during COVID-19 [70]. Third, medical management are urged to find ways to mitigate emotional exhaustion in nurses during COVID-19. For example, counseling and stress management programs, appropriate policies and/or favorable supported systems can be implemented to reduce emotional exhaustion. In addition, education and training focused on resilience, as well as staff feedback sessions, are key interventions for minimizing the risk of burnout for nurses working with COVID-19 patients [71]. Finally, nursing themselves should form the positive attribute and scientific understanding on themselves,through the strengthening to obtain the high self-efficacy. In addition, appropriate relaxation, such as listening to music or exercising, may also be a good way to relieve working stress and reduce turnover intentions when an individual is under work overload.

### Limitations and suggestions

There are at least several limitations that should be addressed. Firstly, it is important to note that there could be other undiscovered variables that may explain why psychological empowerment is a buffer of turnover intention. For example, the role of employee engagement which has been found to be associated with psychological empowerment and job satisfaction [72]. Indeed, future research should include more factors to provide a more comprehensive picture of the psychological empowerment-turnover intention linkage. In addition, self-reported scales were used in this study which may incur problems such as social desirability and psychological defensiveness (such as a more positive attitude towards psychological empowerment or overestimating job satisfaction), and the findings are prone to mono-method bias.

An experimental design or implicit testing should be considered in future research.

Then, results of the hypothesized serial multiple mediation model were based on analyses of cross-sectional data at a single time point. This rendered it difficult to establish directionality and causality [73]. Future researchers are encouraged to use the longitudinal study to explore the causal relationship among the factors. Lastly, the model was tested on a sample of Chinese nurses through convenient sampling,which made it difficult to be universal. Therefore, more studies are needed to examine whether these results can be generalized to nurses in other countries and other samples, and future study need to balance sex ratio.

## Conclusion

Under the background of a global nursing shortage during COVID-19 epidemic, measures to reduce nursing staff turnover intention and improve the job satisfaction of nursing occupation, such as relieving emotional exhaustion among nurses, become an urgent task. This study found that job satisfaction and emotional exhaustion independently mediated the effect of psychological empowerment on turnover intention. Moreover, psychological empowerment was associated with turnover intention through the chain mediating effect of “job satisfaction → emotional exhaustion”. In view of these findings, it is necessary for hospital administrators to enhance the psychological empowerment and job satisfaction levels, and reduce the emotional exhaustion and turnover intention levels among nurses.

## Supplementary Information

**Additional file 1.**

## Data Availability

The datasets generated and/or analysed during the current study are not publicly available due to the authors are still working on them but are available from the corresponding author on reasonable request.
