# Supplementary material for: The mediating effect of job satisfaction and emotional exhaustion on the relationship between psychological empowerment and turnover intention among Chinese nurses during the COVID-19 pandemic: a cross-sectional study
Source: BMC Nurs. 2023 Jun 27;22:221. doi: 10.1186/s12912-023-01357-y (PMC10294374; doi:10.1186/s12912-023-01357-y)
Supplement: Supplementary file 1 — Additional file 1. [file 12912_2023_1357_MOESM1_ESM.docx]

**Appendix 1.**

Here are some statements about your working experience. Please use your own experience, rather than what you think to choose the number that best suits your situation.

1. **Psychological Empowerment Scale**

| Complete  disagreement | Disagreement | Not  necessarily | Agreement | Complete  agreement |
| --- | --- | --- | --- | --- |
| 1 | 2 | 3 | 4 | 5 |

- What I have done at work is meaningful to me.
- I can decide for myself how to do my work.
- I have a great influence on what happened in my organization.
- I have mastered all the skills needed to do my job.
- What I do at work is very meaningful to me personally.
- I have a lot of independence and autonomy in how to complete the work.
- I believe that I have the ability to do everything well in my work.
- I have a great control over what happens in the department.
- My work is very important to me.
- I have a lot of autonomy over how to do my job.
- I am very confident in my ability to complete the work.
- I had a significant impact on what happened in the department.

1. **Turnover Intention Scale**

| never | Very rarely (times a year or less) | Occasionally (once a  month or less) | Often (several times a month) | Frequent (once a week) | Very frequent (several times a week) | every day |
| --- | --- | --- | --- | --- | --- | --- |
| 1 | 2 | 3 | 4 | 5 | 6 | 7 |

- Recently, I’ve been thinking about resigning
- If I had the chance, I would not choose my present job
- If possible, I would consider changing my work

1. **Emotional Exhaustion Scale**

| never | Very rarely (times a year or less) | Occasionally (once a month or less) | Often (several times a month) | Frequent (once a week) | Very frequent (several times a week) | every day |
| --- | --- | --- | --- | --- | --- | --- |
| 1 | 2 | 3 | 4 | 5 | 6 | 7 |

- Work makes me feel physically exhausted.
- Working all day is really tired for me.
- I felt exhausted after work.
- I feel very tired when I get up in the morning and have to face the day's work.
- Work makes me feel like I'm about to collapse.

1. **Job Satisfaction Scale**

| Complete  dissatisfied | Dissatisfied | Not  necessarily | Satisfied | Complete  satisfied |
| --- | --- | --- | --- | --- |
| 1 | 2 | 3 | 4 | 5 |

- Are you satisfied with the intensity and pressure of your job?
- Are you satisfied with the person who guides you (or your leader)?
- Are you satisfied with the relationship with colleagues?
- Are you satisfied with salary of your job?
- Are you satisfied with promotion and development opportunities of your job?
- Given every aspect of your work, are you satisfied with your current work situation?
